# Biofibrous nanomaterials for extracting strategic metal ions from water

**DOI:** 10.1002/EXP.20220050

**Published:** 2022-07-11

**Authors:** Weihua Zhang, Xinpeng Che, Danfeng Pei, Xiaofang Zhang, Yijun Chen, Mingjie Li, Chaoxu Li

**Affiliations:** ^1^ Group of Biomimetic Smart Materials Qingdao Institute of Bioenergy and Bioprocess Technology Chinese Academy of Sciences & Shandong Energy Institute Qingdao China; ^2^ Center of Material and Optoelectronics Engineering University of Chinese Academy of Sciences Beijing China

**Keywords:** adsorption, biological nanofibrils, filtration, metal recovery

## Abstract

Strategic metals play an indispensable role in the related industries. Their extraction and recovery from water are of great significance due to both their rapid consumption and environmental concern. Biofibrous nanomaterials have shown great advantages in capturing metal ions from water. Recent progress in extraction of typical strategic metal ions such as noble metal ions, nuclear metal ions, and Li‐battery related metal ions is reviewed here using typical biological nanofibrils like cellulose nanofibrils, chitin nanofibrils, and protein nanofibrils, as well as their assembly forms like fibers, aerogels/hydrogels, and membranes. An overview of advances in material design and preparation, extraction mechanism, dynamics/thermodynamics, and performance improvement in the last decade is provided. And at last, we propose the current challenges and future perspectives for promoting biological nanofibrous materials toward extracting strategic metal ions in practical conditions of natural seawater, brine, and wastewater.

## Graphical abstract

Extracting strategic metals from water has attracted much attention in the last decade due to their indispensable role and growing demand in the related industries. This review summarizes the research progress in the adsorption of typical strategic metal ions such as Au^3+^, U(VI), and Li^+^ using materials based on biofibrous nanomaterials. The current challenges and future perspectives have been also proposed.

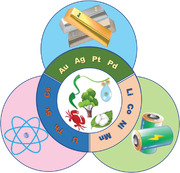

## 1 INTRODUCTION

### 1.1 Scope of strategic metals

Strategic metals are typically identified as those that are vital to high technologies, modern industries, economy development, and military security, whereas they are limited by their supplies because of large consuming amounts, low natural abundance, poor terrestrial deposits, or pressing exhaustion.^[^ ^1^ ^]^ Their identification and categorization have been varying with different regions and times.^[^ ^1^ ^]^

For the past centuries, noble metals, such as gold (Au), silver (Ag), platinum (Pt), and palladium (Pd), have undoubtedly been accepted as strategic metals due to their indispensable roles in jewelry, decoration, catalysis, and electronics.^[^ ^2^ ^]^ For example, in 2020 the total global demand for Au, Ag, Pt, and Pd is about 4020, 25,400, 208, and 302 t, respectively. In electronics alone, the amounts of Au and Ag are up to 250 and 12,800 t^[^ ^3^ ^]^ and are still growing by ∼7–15% per year.^[^ ^4^ ^]^ But the global mine productions of Au, Ag, Pt, and Pd are only about 3000, 25,000, 180, and 210 t, respectively.^[^ ^4^, ^5^ ^]^

With the rapid development of Li‐ion batteries and other energy‐storage devices,^[^ ^1^, ^6^ ^]^ the metals of lithium (Li), cobalt (Co), manganese (Mn), and nickel (Ni) have come into the scope. In particular, with wide applications in ceramics, glass, lubricants, and batteries, the Li demand is estimated to grow by approximately 8–11% annually.^[^ ^2^, ^7^ ^]^ And the Li demand for Li‐ion batteries will double from its 35% share in 2015 to about 70% in 2025.^[^ ^2b^ ^]^ In 2030, the demand for Li carbonate equivalent will reach 1.6 × 10^6^ t,^[^ ^7^, ^8^ ^]^ and there will be a worldwide “lithium rush.”^[^ ^1b^ ^]^

Besides Li, nuclear metals and their isotopes, such as uranium (U), thorium (Th), cesium (Cs), and strontium (Sr), have also attracted great attention due to their roles in the nuclear industry, national defense, agricultural breeding or medical treatment.^[^ ^2^, ^9^ ^]^ For China, nuclear power generation needs about 1.15 × 10^4^ t of U resources every year. Nevertheless, the proven and minable U reserves in China are about 12.9 × 10^4^ t estimated by Organization for Economic Cooperation and Development. In 2018, 88% of U fuel in China was imported.^[^ ^10^ ^]^ As for the whole world, the land reserve of U is expected to be exhausted within 100 years.^[^ ^11^ ^]^

### 1.2 Strategic metals in water

Ocean occupies about 71% of the global surface area and contains 97% of the total water on the earth. And most of the strategic metal ions could be found in the ocean (Table 1). For instance, the average concentration of Li^+^ in the ocean is about ∼0.18 ppm.^[^ ^14^ ^]^ And the amount of overall lithium inventory in the ocean is reported to be approximately 2.6 × 10^11^ t,^[^ ^6b^ ^]^ in stark contrast to that in the terrestrial ores ∼16.7 × 10^6^ t.^[^ ^15^ ^]^ Uranium exists in seawater with an average concentration of ∼3 ppb.^[^ ^12^ ^]^ The available uranium resource reserved in the ocean is up to 4.5 × 10^9^ t, being about 10^3^ times of those in terrestrial uranium ores.^[^ ^16^ ^]^ Besides seawater, high concentrations of Li and U also exist in other water bodies.^[^ ^12^, ^14^, ^17^ ^]^ For instance, the Li concentration reaches 100–1000 ppm in brine water, and 100 ppm in the water produced by oil and gas extraction.^[^ ^14b^ ^]^ And the uranium concentration was confirmed to be 18.3 ppb in Qinghai Lake^[^ ^18^ ^]^ and 1351 ppb in Zabuye Lake.^[^ ^19^ ^]^

**TABLE 1:** Concentrations of various strategic metals in seawater

Element	Concentration in natural seawater (ppb)	Ref.
U	3.1	[12]
Cs	0.2926	[13]
Sr	7885.8	[13]
Li	180	[13]
Ni	0.4696	[13]
Co	0.00236	[13]
Mn	0.0165	[13]

Wastes from electronics, catalysts, automotives, batteries, and nuclear industries are another aquatic resource for the extraction of strategic metals via the hydrometallurgical method.^[^ ^20^ ^]^ A large amount of Au and Pd has been extracted aqueously from electronic wastes,^[^ ^21^ ^]^ for example, discarded computers, mobile phones, and household electronics. There are ∼200–250 g t^−1^ Au and ∼80 g t^−1^ Pd in computer motherboards, and ∼350 g t^−1^ Au and ∼130 g t^−1^ Pd in mobile phone handsets.^[^ ^22^ ^]^ Meanwhile, the used automotives also contain plenty of strategic metals. In automotive catalytic converters, there exists up to 2000 g t^−1^ of Pt group metals in the ceramic catalyst bricks,^[^ ^23^ ^]^ even much higher than the content of Au or Pt group metals in primary ores (on average <10 g t^−1^). One ton of Li metal could be extracted from 8 tons of Li‐ion battery (about 256 used electric vehicle Li‐ion batteries),^[^ ^24^ ^]^ in contrast to ∼250 t of the mineral ore spodumene. In nuclear industries, uraniferous wastewater contains excessive contents of uranium up to 10^2^ ppm.^[^ ^25^ ^]^ Its radioactivity will pose a severe threat to human health (e.g., damage to the liver and kidneys, and even death) and ecological environments.^[^ ^26^ ^]^ According to the standard of the World Health Organization, hexavalent uranium is regarded as a carcinogen, and its concentration in water should not exceed 30 ppb.^[^ ^27^ ^]^ Therefore, extracting strategic metals from the eluents of these wastes and wastewater of the related industries should be also an important way for the recycling of strategic metals.

### 1.3 Biofibrous nanomaterials as absorbents

In principle, efficient extraction of metal ions from water is challenged by their low concentrations (e.g., ∼0.18 ppm Li^+^ in the ocean), multivalent existent forms (e.g., hexavalent UO_2_^2+^ at pH ≤ 4 and [(UO_2_)*_x_* (OH)*_y_*]^2^ *^x^* ^−^ *^y^* in pH > 7), complex coexisting species (e.g., Cu, Ni, Pb, Sn, Mn, Ag, Au and Pd ions in electronic waste leachate) and harsh conditions (e.g., high acidity and salinity).^[^ ^2^, ^9^, ^26^, ^28^ ^]^ Many approaches have been used to extract certain metal ions, including chemical precipitation, solvent extraction, evaporative recovery, reverse osmosis, etc.

Among these extraction approaches, adsorption attracted particular interest due to its low cost, convenient operation, low risk of secondary pollution, and adsorbent recyclability.^[^ ^26b^ ^]^ A variety of absorbents (e.g., carbon materials of activated carbon and graphene, inorganic materials of metal oxides and molecular sieves, metal/covalent‐organic frameworks, and organic polymers of ion exchange resins) have been endeavored to achieve large adsorption capacity, high selectivity and fast dynamics in capturing aquatic metal ions.^[^ ^26^, ^28^, ^29^ ^]^ Nanostructured materials, such as graphene, carbon nanotubes, oxide nanoparticles, and zeolite‐based nanosorbents, have been extensively investigated as adsorbents owing to their larger specific surface area, super binding affinities, unique nanoscale effects, and interfacial phenomena.^[^ ^9^, ^26^ ^]^

Biologic nanofibrils are typically identified as biologic fibrous nanomaterials with a diameter of < 100 nm and a length of >10^2^ nm,^[^ ^30^ ^]^ such as cellulose nanofibrils from wood, chitin nanofibrils from crab shells, fibroin nanofibrils from silk and collagen nanofibrils from animal ligaments.^[^ ^30^, ^31^ ^]^ As an emerging class of nanomaterials and absorbents,^[^ ^9^, ^29^, ^31^, ^32^ ^]^ biologic fibrils not only have intrinsic biomass advantages (e.g., natural abundance, sustainability, biodegradability, and biocompatibility), but also have the advantages of large aspect ratios, high mechanical strength, solvent dispersibility and flexibility of chemical modification. Therefore, compared with the traditional nanostructured adsorbents, biologic fibrils afforded great promise for high adsorption capacity, fast dynamics, low cost, environmental friendliness, good processability, and high structural stability. Their functional groups and nanoscale architecture enabled them to extract metal ions via electrostatic attraction, chelation, chemical reduction,^[^ ^33^ ^]^ and nano‐sieving.^[^ ^34^ ^]^ Different forms (e.g., aerogels, beads in stationary beds, and membranes for filtration^[^ ^16^, ^35^ ^]^) were fabricated to achieve high porosity, mechanical stability, and antifouling properties.

These nanofibrils could be produced through either a “bottom‐up” route of supramolecular self‐assembly,^[^ ^30^, ^32^ ^]^ or a “top‐down” exfoliation approach from natural biomaterials. In a typical exfoliation procedure, chemical modifications (e.g., 2,2,6,6‐tetramethylpiperidine‐1‐oxyl radical (TEMPO)‐mediated oxidation, deacetylation, and partial acidolysis) and mechanical treatments (e.g., ball milling, high‐pressure homogenization, microfluidization, and ultrasonication) were used to relieve the interactions between nanofibrils (e.g., van der Waals force and hydrogen bonding). Their intrinsic hydroxyl, carboxyl, and amine groups further enabled chemical modification (e.g., oxidation, esterification, etherification, and amidation^[^ ^30^, ^32^ ^]^) to introduce specific ligands for selectively binding metal ions (e.g., amidoxime toward U(VI), sulfhydryl toward Au(III) and crown ether toward Li^+[^ ^16^, ^36^ ^]^).

**FIGURE 1:**
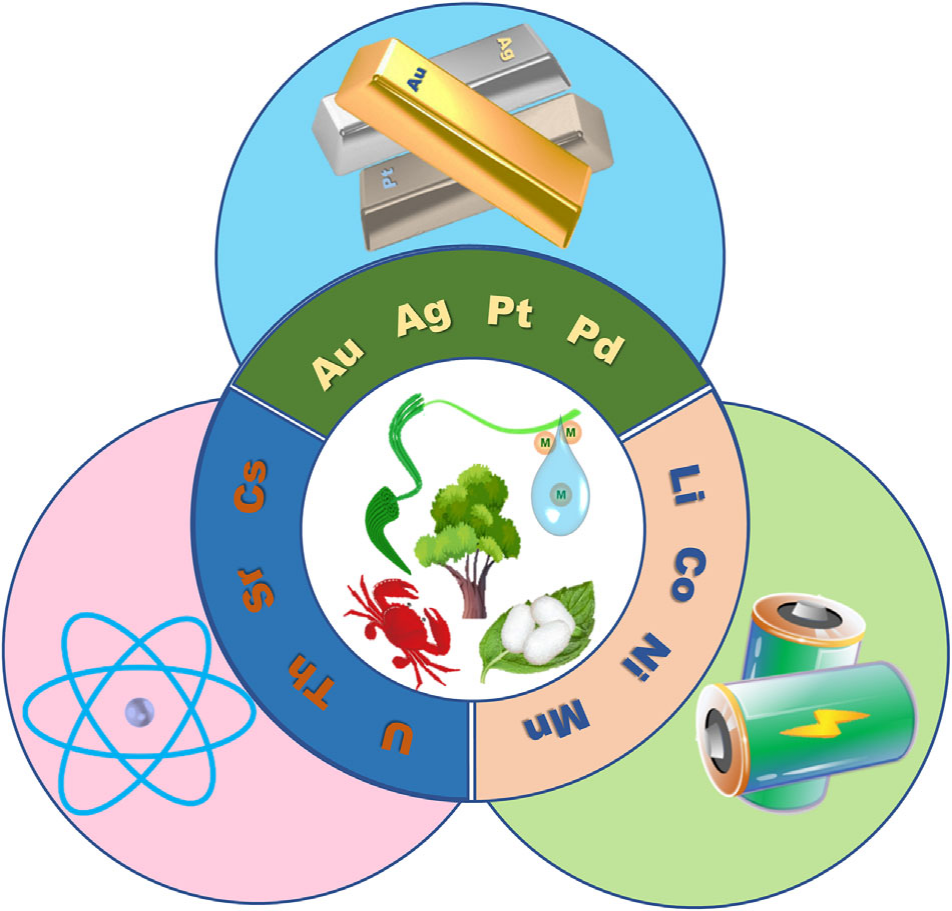
Extracting typical strategic metal ions with bio‐fibrous nanomaterials

In the past years, some specialized reviews have summarized the preparation, chemical modification, and applications of biological nanofibrils.^[^ ^30^, ^31^, ^37^ ^]^ There were also several environmental reviews about removing the pollutants of heavy‐metal ions (e.g., Cr(VI), As(V), Pb^2+^, Cd^2+^, Hg^2+^, and Cu^2+^) from wastewater with individual type of biological nanomaterials like protein nanofibrils, nanocellulose, and electrospun nanofibrous carbohydrates.^[^ ^29^, ^31^, ^32^, ^36^, ^38^ ^]^ In this review, the comprehensive progress is detailed about three types of typical bio‐fibrous nanomaterials (i.e., cellulose, chitin, and protein nanofibrils) in extracting typical strategic metal ions, including noble metal ions (e.g., Au(III), Ag^+^ and Pt^2+^), nuclear metal ions (e.g., U(IV), Th(IV) and Cs^+^) and Li‐battery related metal ions (e.g., Li^+^, Ni^2+^, and Co^2+^), as illustrated in Figure 1. The current challenges, possible solutions, and future perspectives are also provided.

## 2 EXTRACTION OF NOBLE METALS WITH BIOFIBROUS NANOMATERIALS

Recovery of noble metals is known to be of importance for the sake of the economy and environment. As non‐renewable resources, most of the noble metals are unabundant on the earth but indispensable in many fields. When their aqueous wastes (e.g., from electronics and catalysts^[^ ^21^ ^]^) entered the environment, it resulted in many ecological and health disasters.^[^ ^3b^ ^]^ The procedures of solvent extraction,^[^ ^39^ ^]^ membrane separation electrolysis,^[^ ^40^ ^]^ electrochemical processes,^[^ ^41^ ^]^ and adsorption^[^ ^42^ ^]^ have been reported to recover noble metals. Biofibrous nanomaterials were used to extract noble metals via static adsorption, dynamic filtration, and chemical reduction.

### 2.1 Static absorption

Biological nanofibrils have been developed widely for precious metal ions capture due to their abundant active groups and fibrous morphology. For example, Jafari et al.^[^ ^43^ ^]^ found TEMPO‐oxidized cellulose nanofibrils could recover precious Au ions from chloride solution with a highest adsorption ability of 15.4 mg g^–1^. Mathew's group^[^ ^44^ ^]^ reported the potential of cellulose nanocrystals and cellulose nanofibrils isolated from bio‐residues to adsorb Ag^+^ from wastewater. Cellulose nanocrystals exhibited a higher extracting efficiency of 64% at neutral pH, ∼37% higher than that of cellulose nanofibrils due to the higher negative surface charge on the cellulose nanocrystals.

**FIGURE 2:**
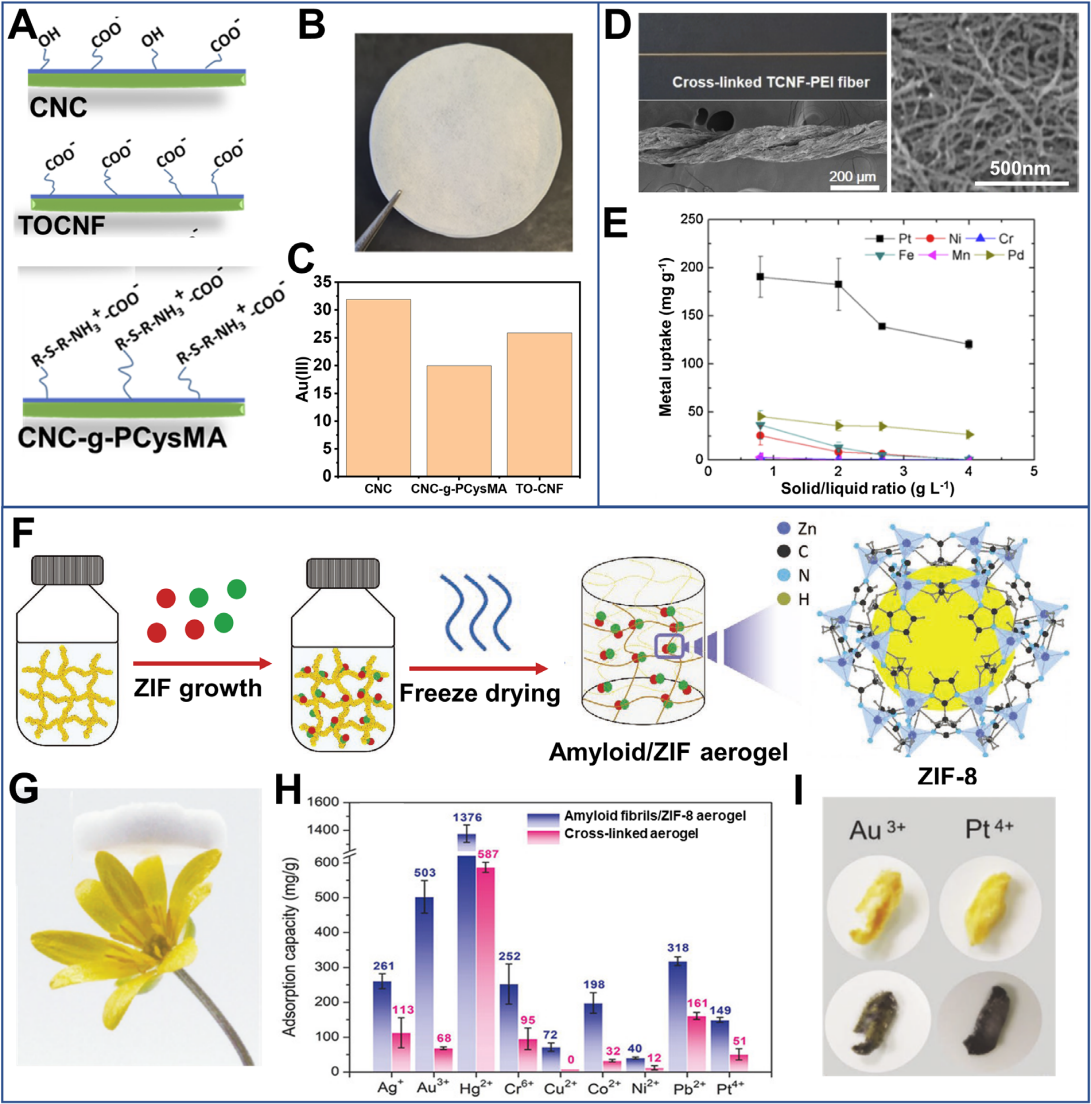
(A) Schematic depiction of cellulose nanocrystalline (CNC) with different functional groups and (B) optical images of the membranes (51 mm diameter). (C) The Au ions adsorption capacity of different nanocellulose membranes. TOCNF: TEMPO (2,2,6,6‐tetramethylpiperidine‐1‐oxyl radical)‐mediated oxidized cellulose nanofibers and CNC‐g‐PcysMA: Zwitterionic polymer grafted cellulose nanocrystals. Reproduced with permission.^[^ ^45^ ^]^ Copyright 2021, Elsevier Ltd. (D) Optical and scanning electron microscope (SEM) images of neat cross‐linked tunicate cellulose nanofibrils modified with polyethylenimine (TCNF‐PEI). (E) Metal ion uptake performance of TCNF‐PEI. Reproduced with permission.^[^ ^35d^ ^]^ Copyright 2019, Elsevier Ltd. (F) Graphic illustration of the preparation of amyloid/ZIF‐8 hybrid aerogel. (G) Optical image of the hybrid aerogel on top of wildflower. (H) Maximum uptake of different metal ions by amyloid/ZIF‐8 hybrid aerogel and pure amyloid aerogel. (I) The influence of 0.1 M ethylenediaminetetraacetic acid (EDTA) solution on the hybrid aerogels loaded with metal ions. Reproduced with permission.^[^ ^34d^ ^]^ Copyright 2021, John Wiley and Sons

Their absorbing properties may be further tuned by chemical modifications. By combining zwitterionic cellulose nanocrystals and TEMPO‐oxidized cellulose nanofibrils in Figure 2A–C, Georgouvelas^[^ ^45^ ^]^ produced the fibrous membranes to capture Au ions with a capacity of ∼15.4 mg g^–1^. Enzymatic phosphorylated cellulose nanocrystals prepared by Liu et al.^[^ ^46^ ^]^ showed a higher adsorption selectivity to Ag^+^ compared with typical competing ions of Cu^2+^ and Fe^3+^. Hong's group^[^ ^47^ ^]^ cross‐linked polyethyleneimine‐modified cellulose nanofibrils by glutaraldehyde (Figure 2D). The resultant aerogel presented excellent Pt^2+^ adsorption capacity (∼600 mg g^–1^), and high selectivity toward Pt^2+^ in the presence of other metals (e.g., Pd^2+^, Cr^6+^, Mn^2+^, Fe^3+^, and Ni^2+^) (Figure 2E). Ruan et al.^[^ ^48^ ^]^ prepared cysteine modified 2,3‐dialdehyde cellulose nanofibrils (originating from the green algae *Cladophora*) via reductive amination for adsorption of palladium ions. The adsorbent presented a Pt^2+^ extraction capacity of 130 mg g^–1^ and high adsorption kinetics (80% of maximum capacity was accomplished within 2 h).

The extraction properties of biologic fibrils could be further enhanced by biomimetic mineralization of metal‐organic framework (MOF) (Figure 2F,G). Raffaele et al.^[^ ^49^ ^]^ showed that the amyloid fibrils with the coating of 2‐methylimidazole zinc salt (ZIF‐8) could effectively adsorb Au ions (503 mg g^−1^) (Figure 2H,I). A decay in adsorption efficiency at low pH values was possibly due to electrostatic repulsion arising from the protonation of amyloids and ZIF‐8 preventing metal ions from approaching the active sites. In general, static adsorption can effectively extract strategic metal ions at both high and low concentrations. However, the static adsorption usually has a slow mass transfer process, which results in a long extraction time.

### 2.2 Continuous filtration

**FIGURE 3:**
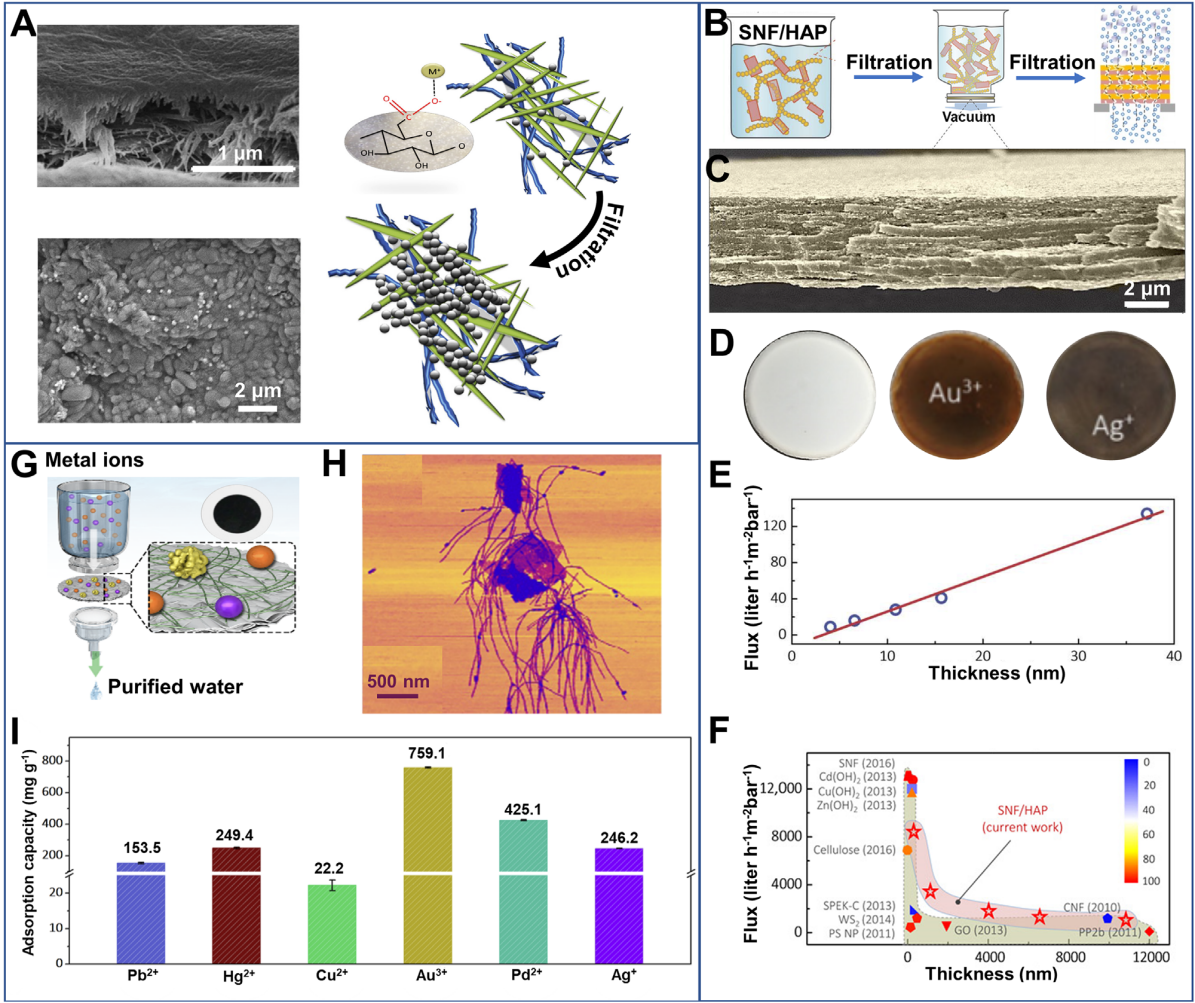
(A) Illustration of nucleation effect of metal ions during filtration. The SEM images confirm the nucleation on the surface of membranes before and after filtration. Reproduced with permission.^[^ ^33d^ ^]^ Copyright 2016, Elsevier Ltd. (B) Dispersions of silk nanofibrils (SNF) and hydroxyapatite (HAP) were filtrated into membranes for metal ions extraction. (C) The cross‐sectional SEM image of the hybrid membrane, which shows nacre‐like and highly ordered multilayer structures. (D) Image of the hybrid membrane before and after flux‐controllable filtration. (E) The relationship of film thickness and filtration flux. (F) The relationship between thickness and water permeability of the hybrid membrane and other membranes in literature. Reproduced with permission.^[^ ^34e^ ^]^ Copyright 2017, American Association for the Advancement of Science (AAAS). (G) Mechanism for extracting metal ions from water. (H) Atomic force microscope (AFM) image of the nanocomposites of molybdenum disulfide (MoS_2_) and SNF (weight ratios: 1:4). (I) Extraction performance of the hybrid membranes for different metal ions. Reproduced with permission.^[^ ^34c^ ^]^ Copyright 2020, American Chemical Society

Biologic nanofibrils could be easily possessed into membranes by filtration or deposition. With sufficient absorption kinetics and capacity, the membranes have been designed to capture precious metal ions through continuous filtration. One of the most frequently used types of biologic fibrils was nanocellulose. By grafting with sulfate or carboxyl surface groups and treating with acetone,^[^ ^33d^ ^]^ the membranes of cellulose fibrils exhibited a tensile strength at break of 95 MPa in the dry state and 3.7 MPa in the wet state. A water flux of 25 L m^–2^ h^–1^ was achieved at a pressure differential of 0.45 MPa with an exceptional Ag^+^ capturing efficiency near 100% (Figure 3A). When supporting cellulose nanocrystals on cellulose microfiber sludge,^[^ ^50^ ^]^ the bilayer membranes showed a water permeability as high as 900 to 4000 L h^–1^ m^–2^ at < 1.5 bars. Approximately 94% Ag^+^ was trapped when filtrating industry effluent with trace Ag^+^ (0.01 mg L^–1^).

The membranes of β‐lactoglobulin amyloid fibrils were also employed by Mezzenga^[^ ^51^ ^]^ to capture Ag^+^ and Pt^2+^ through continuous filtration, due to the strong binding affinity of amino residues. When further combing activated carbon,^[^ ^52^ ^]^ the hybrid membranes could decrease the concentration of Au ions from 30 ppm to 0.105 ppm by filtration. Even for KAu(CN)_2_ solution with a high concentration (561 ppm), one cycle of filtration could decrease its concentration to 0.134 ppm with an adsorption efficiency of 99.98%. In analog, silk nanofibrils, as a kind of low‐cost protein nanomaterial, can be also developed for fibrous membrane fabrication to capture metal ions by filtration (Figure 3B–I). For example, Ling^[^ ^34e^ ^]^ combined silk nanofibrils with hydroxyapatite nanoplates into hybrid membranes (Figure 3B–D). The thickness of 0.3 µm offered a fast transportation with 8355 L m^–2^ h^–1^ bar^–1^ (Figure 3E,F). Following the chelation and ion exchange mechanism, the thickness of 4 µm showed the maximum adsorption capacities of 164.2, 132.5, and 145.8 mg g^–1^ for Au(III), Ni^2+^ and Cr^3+^, respectively. Although membrane filtration is an effective method to extract noble metals, there still have several concerns for practical applications especially in complex environments such as membrane preparation process, structural stability, porosity tuning, and anti‐fouling properties.

### 2.3 Chemical reduction

Functional groups of biologic fibrils may also enable chemical reduction and capture of noble metal ions. By adding sulfonate groups to nanocellulose, Dwivedi et al.^[^ ^53^ ^]^ observed a good Au (III) adsorption capacity (∼60 mg g^–1^) even at low pH (∼2) from Au‐containing wastewater, in which Au ions could be reduced into Au nanoparticles. A maximum regeneration ratio of up to 99% was reached with a solution of 0.5 M thiourea and 1 M HCl as eluent, by forming a stable cationic species of Au in the acidic medium (Au[CS(NH_2_)_2_]_2_^+^).

**FIGURE 4:**
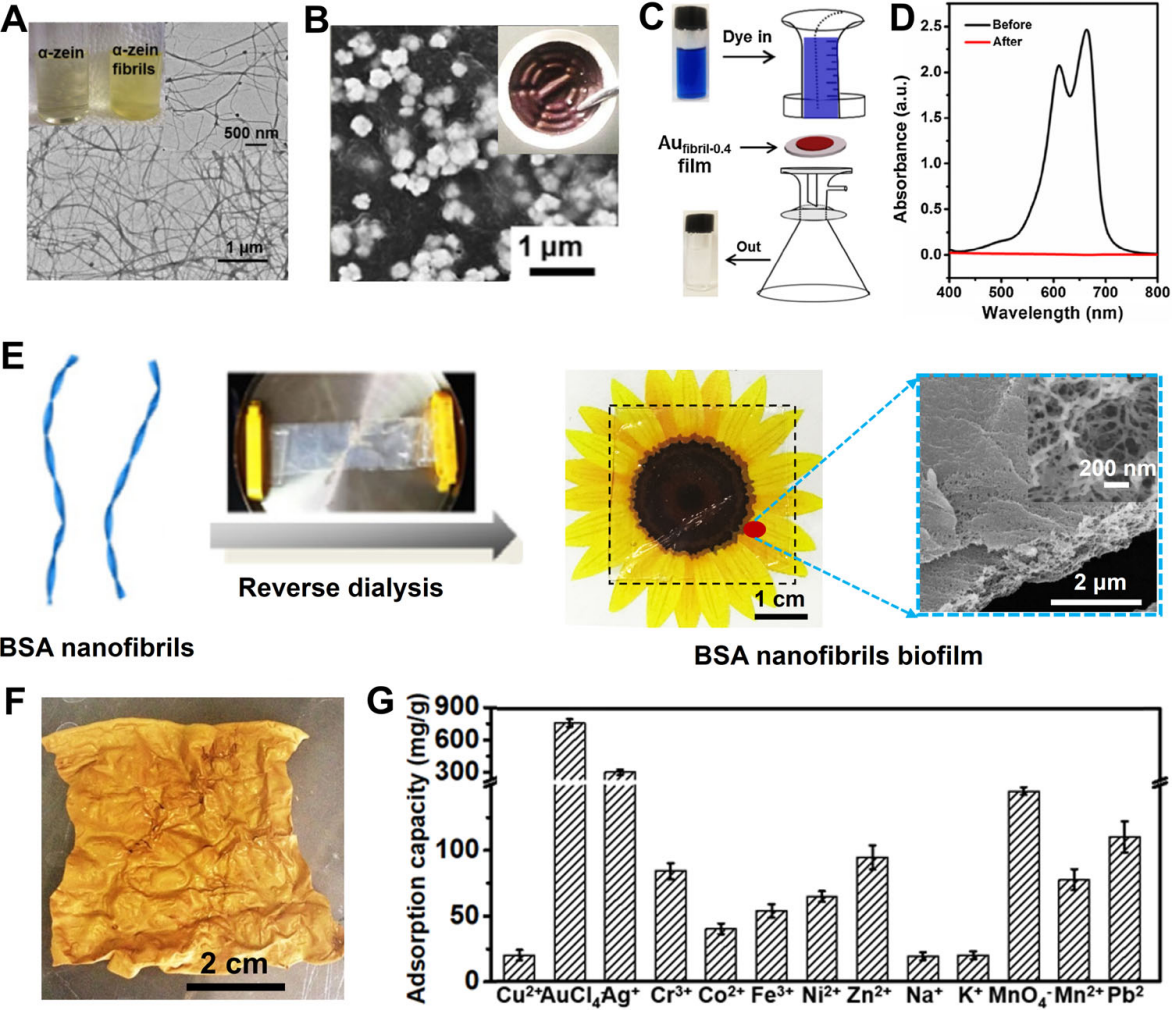
(A) Transmission electron microscope (TEM) image of *α*‐zein nanofibrils. The inset is solution of *α*‐zein and dispersion of its nanofibrils. (B) SEM images of Au nanoparticles deposited on filtering membrane. The inset is the membrane photograph (right top). (C) Illustration of flow catalysis of Au‐deposited membrane for organic dye. (D) UV–vis absorbance of methylene blue before and after passing through the membrane. Reproduced with permission.^[^ ^54^ ^]^ Copyright 2016, Elsevier Ltd. (E) Illustration of the pathway from BSA nanofibrils into biofilms. Visual observation of the transparent biofilm. Thickness: 95 µm. SEM images of typical fibrous microstructures in biofilm. (F) Extracting and reducing AuCl_4_^−^ simultaneously for fabricating conductive hybrid film. (G) Supramolecular biofilms as trapping foams for different metal ions. Reproduced with permission.^[^ ^55^ ^]^ Copyright 2018, Elsevier Ltd

By using nanofibrils of *α*‐zein as the reductant, capping agent, and stabilizer (Figure 4A),^[^ ^54^ ^]^ we captured and reduced Au ions into nanocrystals as the catalysts for the degradation of organic dyes and glucose detection (Figure 4B–D). Through a reverse dialysis procedure (Figure 4E),^[^ ^55^ ^]^ we further produced supramolecular biofilms from amyloid fibrils of bovine serum albumin (BSA). These nanofibrils exhibited excellent absorption capacities to AuCl_4_^−^ (765 mg g^–1^) and Ag^+^ (305 mg g^–1^) because of –S–S– and –SH groups in BSA molecules as well as their reducing effect (Figure 4F).^[^ ^52^, ^56^ ^]^ These capacities were much higher than those of other metal ions such as Cu^2+^, Mn^2+^, Cr^3+^, Ni^2+^, Pb^2+^, Co^2+^, Fe^3+^, Na^+^ and K^+^ (Figure 4G).^[^ ^55^ ^]^

**FIGURE 5:**
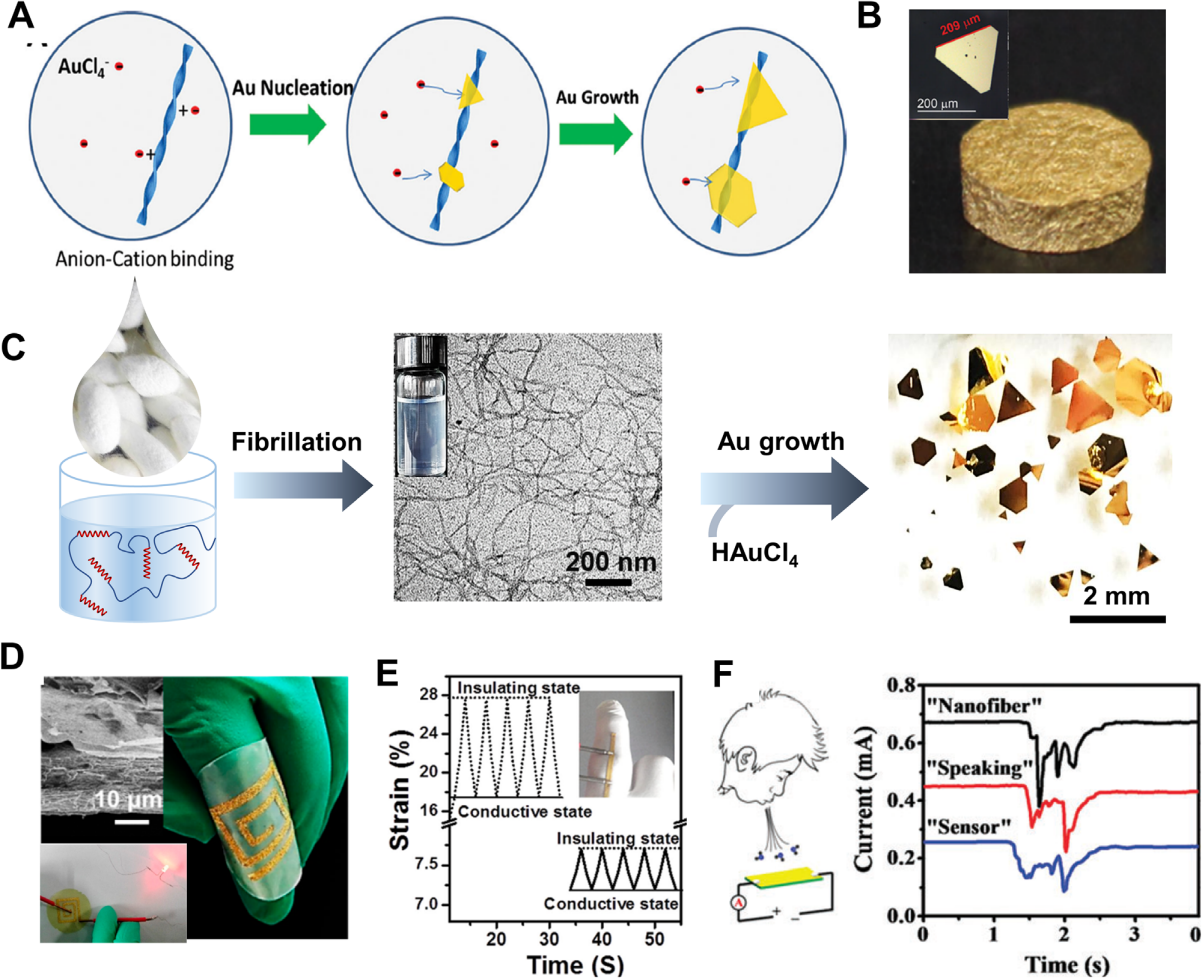
(A) Illustration of the pathway followed to prepare macroscopic single‐crystal gold microflakes from chloroaurate ions and amyloid fibrils. Reproduced with permission.^[^ ^61^ ^]^ Copyright 2015, John Wiley and Sons. (B) Photograph of the gold aerogel. The inset gives single‐crystal gold microflakes observed with TEM. Reproduced with permission.^[^ ^33e^ ^]^ Copyright 2020, John Wiley and Sons. (C) The pathway to prepare millimetric Au crystals: Regeneration of silk fibroin solution, silk fibroin fibrillization induced by ethanol, and reduction of AuCl_4_^−^ with silk fibroin nanofibrils. Reproduced with permission.^[^ ^58^ ^]^ Copyright 2018, American Chemical Society. (D) Patterning Au microflakes on silk fibroin nanofibrils/amyloid nanofibrils membrane as flexible electronic circuit. The inset presents Au microflakes deposited membrane and the conductive property of flexible electronic circuit. Reproduced with permission.^[^ ^33a^ ^]^ Copyright 2017, American Chemical Society. (E) Strain‐controlled in‐plane conductive and insulating states for different strains 27.5% (dotted line) and 45% (solid line), which can be utilized for sensing finger bending (the inset image). Reproduced with permission.^[^ ^61^ ^]^ Copyright 2015, John Wiley and Sons. (F) The performance of breath sensing evaluation via the electric conductivity during speaking different words due to high humidity‐sensitivity of hybrid circuits Au microflakes deposited on the membrane of chitin nanofibrils. These Au microflakes were prepared with chitin nanofibrils. Reproduced with copyright permission.^[^ ^64^ ^]^ Copyright 2017, Royal Society of Chemistry

As the mild reducing and capping agents, proteins and their assemblies (e.g., *α*‐zein^[^ ^57^ ^]^ and silk nanofibrils^[^ ^58^ ^]^) were found to reduce Au ions into nanowires and platelets in a “kinetically controlled” way.^[^ ^58^, ^59^ ^]^ Single‐crystal Au platelets with a large area (10^4^ µm^2^)^[^ ^60^ ^]^ and high aspect ratio (beyond 10^3^)^[^ ^61^ ^]^ were produced by using amyloid fibrils of β‐lactoglobulin (Figure 5A), being promising to produce functional composites for applications in optical nanocircuitry, single‐crystal electronics, nanoantenna, sensing, catalysis, and imaging.^[^ ^60^, ^61^, ^62^ ^]^ For instance, Au aerogels (18 karats) were produced by Mezzenga,^[^ ^33e^ ^]^ showing a Young's modulus ∼50 MPa and an ultra‐low density of 1.7 g cm^−3^ (Figure 5B), which meet typical requirements of jewelry and watch‐making industry, radiation shielding, catalysis, and electronics.

Due to the unique amino sequence, silk fibrils could assist to produce single‐crystal Au flakes with a lateral dimension exceeding 2 mm (Figure 5C),^[^ ^58^ ^]^ being orders of magnitude larger compared with those synthesized by conventional chemical methods.^[^ ^63^ ^]^ Besides protein fibrils, deacetylated chitin nanofibrils also showed the capability of reducing chloroauric ions and further guiding the growth of Au crystals into different geometries (i.e., nanosheets, nanokites, and nanoribbons).^[^ ^64^ ^]^ Their large planar surfaces also offered a unique platform for microfabrication of devices applicable in catalysis, biomedicine, electronics, sensing, and optics (Figure 5D–F).^[^ ^33^, ^61^, ^64^ ^]^

By combining 2D transition metal dichalcogenide nanosheets (i.e., MoS_2_) and silk nanofibrils, Ping^[^ ^34c^ ^]^ produced the layered membranes to capture ions of noble metal by filtration (Figure 3G,H). The composite membrane adsorbed metal ions from water with negative charges, and in situ reduced them into insoluble nanoparticles. (e.g., capacity: Au(III) ∼759 mg g^−1^ and Pd^2+^ ∼425 mg g^−1^) due to the reducing effect of the MoS_2_ nanosheets (Figure 3I). Chemical reduction may provide a new insight to acquire zero‐valent noble metals and their functional materials from water directly.

## 3 EXTRACTION OF NUCLEAR METALS WITH BIOFIBROUS NANOMATERIALS

Although there is a large amount of nuclear metal ions in the seawater or wastewater, their efficient extraction is still a big challenge because of the complex forms (e.g., hexavalent UO_2_^2+^ in aerobic and acidic conditions, and [(UO_2_)*_x_* (OH)*_y_*]^2^ *^x^* ^−^ *^y^* at high pH values), massive coexisting species (e.g., V(V), Fe^3+^, Ca^2+^, and Mg^2+^), extremely low concentration (e.g., ∼3 ppb in the ocean) and harsh conditions (e.g., ocean salinity ∼3.5 wt%).^[^ ^9^, ^65^ ^]^

### 3.1 Static adsorption of uranium ions

Many functional groups have been revealed to be able to chelate uranium ions in water such as amidoxime, carboxyl, amino, phosphonate, and hydroxy groups,^[^ ^9^, ^65^, ^66^ ^]^ which were either intrinsic of biological fibrils or added to their surfaces for capturing uranium ions.^[^ ^3^, ^32^ ^]^ Cellulose nanofibrils with amidoxime,^[^ ^16^ ^]^ carboxyl,^[^ ^67^ ^]^ and phosphonate^[^ ^68^ ^]^ groups were reported to show a rapid adsorption kinetics and super uranium removal capacity (> 1000 mg g^−1^). For instance, Wang^[^ ^69^ ^]^ crosslinked cellulose nanofibrils with polyamide epichlorohydrin resin and presented a high static uptake of 440 mg g^−1^ with initial concentration at 50 mg L^−1^. Anirudhan^[^ ^70^ ^]^ grafted poly(itaconic acid)‐poly(methacrylic acid) onto cellulose nanocrystals, and further composited them with nanobentonites. The adsorbent reached an extraction ability of 121 mg g^−1^ with the initial U(VI) concentration at 150 mg L^−1^ in the simulated solutions.

**FIGURE 6:**
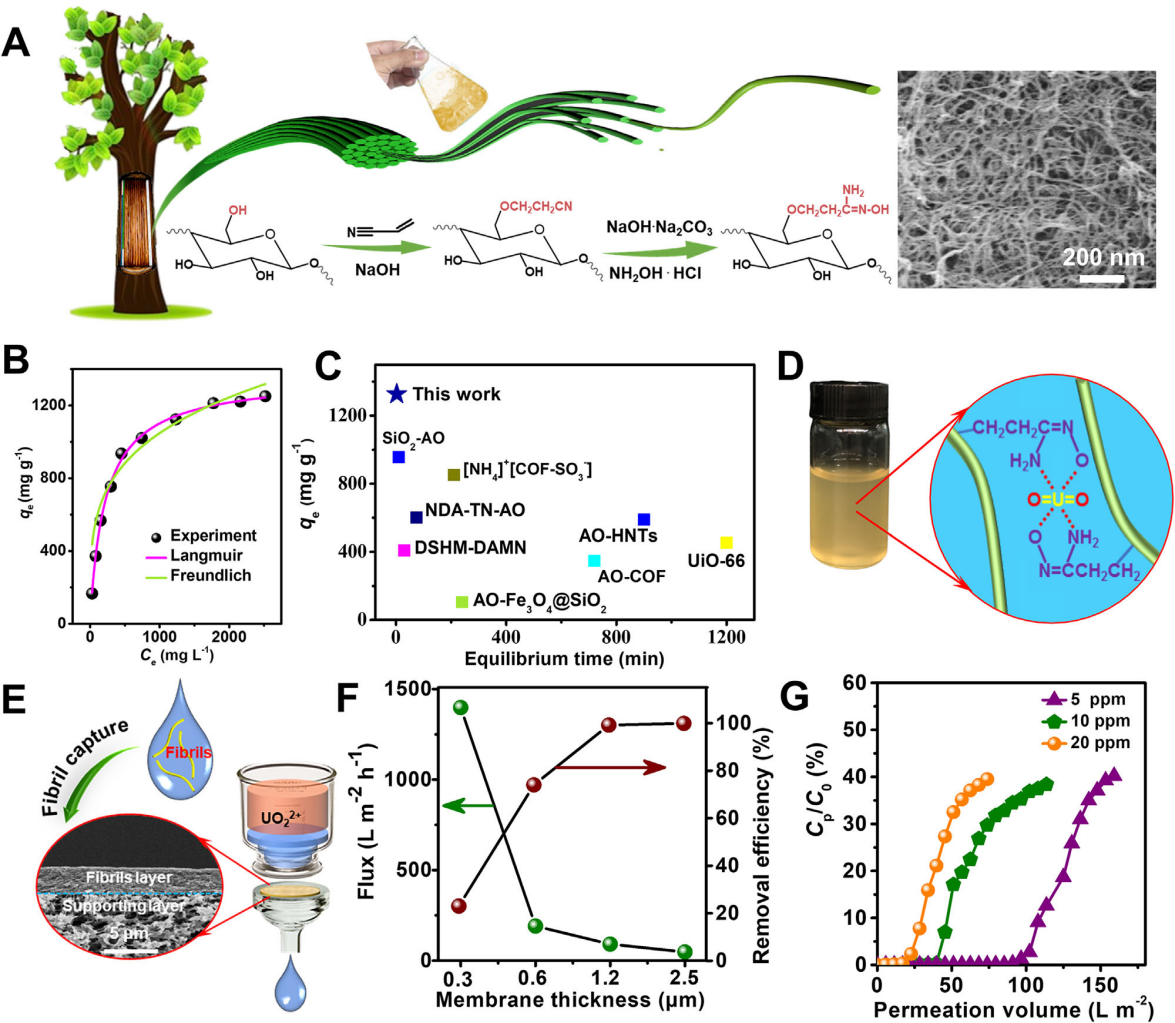
(A) Illustration of process for producing cyanoethylated cellulose fibrils by manual shake and further amidoximation for amidoximated cellulose nanofibrils. SEM image of amidoximated cellulose nanofibrils was given. (B) Langmuir and Freundlich model (pH: ∼8) of the adsorption process. (C) Uranium adsorption performance in comparison with various adsorbents. Amidoxime functionalized silica framework (SiO_2_‐AO); Ammoniating SO_3_H‐decorated covalent organic framework ([NH_4_]^+^[COF‐SO_3_^−^]; Naphthalene‐based sp2‐carbon amidoxime‐functionalized covalent organic frameworks (NDA‐TN‐AO); Diaminomaleonitrile functionalized double‐shelled hollow metal‐organic framework (DSHM‐DAMN); Amidoxime‐functionalized halloysite nanotubes HNTs adsorbents (AO‐HNTs); Amidoxime‐functionalized covalent organic frameworks (AO‐COF); Antibacterial UiO‐66 metal‐organic frameworks (Anti‐UiO‐66); Amidoxime modified Fe_3_O_4_@SiO_2_ (AO‐Fe_3_O_4_@SiO_2_). (D) Chelation of uranyl ions with amidoxime groups. (E) Fabricating amidoximated cellulose nanofibrils membrane via filtration for continuous extracting uranium ions. (F) The relationship of water flux and uranium removal efficiency at different film thicknesses, with uranium concentration of ∼20 ppm. (G) Dependence of uranium concentration (*C*_p_) of filtrate on volume of permeation solution. Reproduced with permission.^[^ ^16^ ^]^ Copyright 2022, Royal Society of Chemistry

Kaur et al.^[^ ^71^ ^]^ prepared amidoximated cellulose nanofibrils by introducing diaminomaleonitrile followed by treating with hydroxylamine hydrochloride. The fibrous adsorbents presented a high uptake capacity (220 mg g^−1^ at pH 7), with a negligible decay after 5 adsorption‐desorption cycles. In addition, the uranium adsorption performance can also be improved by increasing the content of amidoxime groups. Liu^[^ ^34b^ ^]^ produced microspheres of amidoximated cellulose nanofibrils through the sequential process of Michael addition, amidoximation reaction, and phase separation. With a porosity of ∼76%, these fibrous microspheres showed a high adsorption capacity of 517 mg g^−1^ at pH 7. Through a filtration column, an adsorption capacity of 50 mg g^−1^ could be achieved by filtrating simulated seawater with 8 ppm for 24 days. We found an efficient exfoliation method (i.e., manual shake or homogenization for 30 min) to produce cyanoethyl cellulose fibrils with a high conversion of 90% from bleached softwood kraft pulp (Figure 6A).^[^ ^16^ ^]^ After the cyanoethyl groups were converted into amidoxime groups, the obtained amidoximated cellulose nanofibrils (degree of substitution ∼1.58) presented a high adsorption capacity (1327 mg g^−1^) and fast kinetics (< 5 min) in simulated uranium solutions (Figure 6B–D) and a capacity of 9.4 mg g^−1^ after immersing in natural seawater for only 30 days.^[^ ^16^ ^]^

**FIGURE 7:**
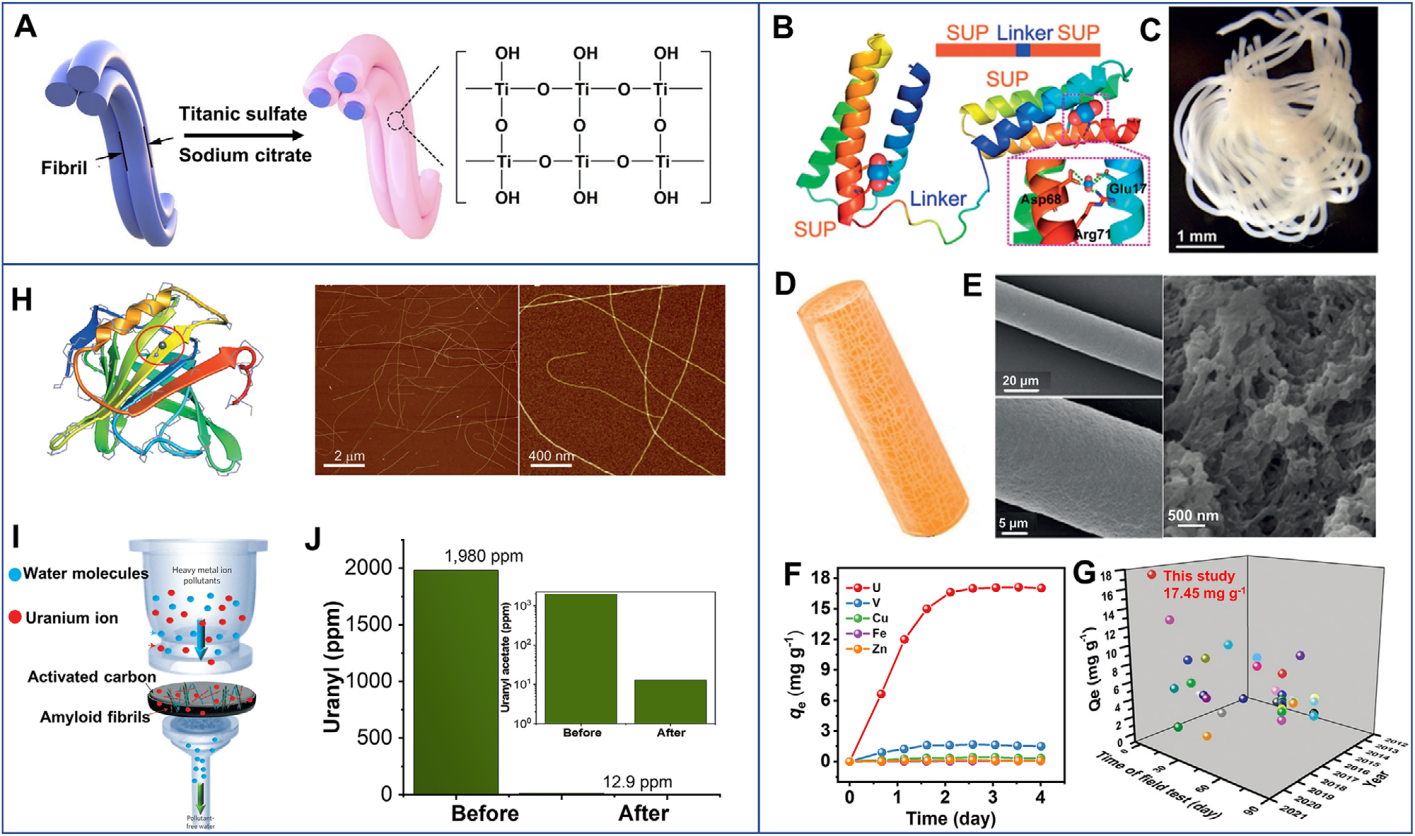
(A) Illustration of immobilization of hydrous titanium oxide onto collagen nanofibers. Reproduced with permission.^[^ ^72^ ^]^ Copyright 2021, Elsevier Ltd. (B) Domain composition and mechanism for uranyl binding of dual superb‐uranyl binding protein (DSUP). (C) Photograph, (D) schematic loofah‐shape, and (E) SEM images at different magnifications of the wet DSUP fibers. (F) Extraction kinetics of DSUP to different metal ions in real seawater. (G) The uranium adsorption capacities in real sweater with different adsorbents in literature. Reproduced with permission.^[^ ^75b^ ^]^ Copyright 2020, John Wiley and Sons. (H) Molecular structure of the β‐lactoglobulin protein and AFM images of the β‐lactoglobulin amyloid nanofibrils. (I) Schematic representation of metal ions extraction by amyloid nanofibrils‐activated carbon hybrids. (J) Concentrations of uranium solution before and after filtration through the hybrid membrane. Reproduced with permission.^[^ ^34f^ ^]^ Copyright 2016, Nature Publishing Group

Collagen nanofibers with hierarchical fibrous morphology and abundant active groups, such as –OH, –COOH, and –NH_2_, also promise in capturing aquatic uranium. Tang's group^[^ ^72^ ^]^ introduced hydrous titanium oxide onto collagen nanofibers (50–200 nm), which presented a capacity of U(VI) adsorption of 328 mg g^−1^ due to the abundant hydroxyl groups of hydrous titanium oxide and amino groups of collagen (Figure 7A).

Among the above groups, amidoxime‐based materials have been considered as one of the most promising candidates for extracting uranium from aqueous systems owing to their unique interactions with uranium ions. The amidoxime group contains oxime nitrogen, amino nitrogen, and oxime oxygen, which can provide electron donors to interact with uranyl ions. In addition, the amphoteric nature of amidoxime group can allow amidoxime‐based adsorbents to extract uranium effectively under a wide pH range.^[^ ^73^ ^]^

In most cases, biologic fibrils were used to extract uranium ions from their artificial solutions. It remains challenging to capture uranium ions in natural seawater, due to the low uranium concentration and the co‐existence of other metal ions. Feng et al.^[^ ^74^ ^]^ utilized natural marine crab carapaces consisting of chitin nanofibrils and crystalline calcium carbonate to extract uranium ions in seawater. This composite adsorbent had a U(VI) extraction capacity of 1.38 mg g^−1^ due to its coordination with uranyl ions.

Yuan^[^ ^75^ ^]^ designed a superb‐uranyl binding protein by genetic engineering and further produced it into protein fibers (Figure 7B–E). With outstanding mechanical properties, high selectivity, and remarkable resistance against biofouling, the fibers showed an exceedingly fast saturation speed (within 3 days) with the highest uranium uptake capacity of ∼17.5 mg g^−1^ in seawater (Figure 7F), both of which are superior to other adsorbents (Figure 7G).^[^ ^12^, ^16^, ^76^ ^]^ In addition, the recombinant protein fibers also demonstrated high selective extraction for uranium ions against the other interfering co‐existing metal ions in real seawater. Eluted with 100 mM ethylenediamine tetraacetic acid, the fibers could maintain ∼72.4% of the initial uranium adsorption capacity after being reused for 8 cycles.

By using carbon nanotubes as the photo‐thermal agent, Luo^[^ ^77^ ^]^ further used photothermal evaporation to enhance the adsorption capacity of cellulose fibrils. The solar‐powered “pump” enhanced the adsorption capacity to 46 µg g^−1^ in 1 ppm U(VI) spiked seawater, being ∼48.6% higher than that without irradiation. This is because the local solar‐heating effect increased the adsorption rate and capacity of U ions.

### 3.2 Dynamic filtration of uranium ions

As an efficient separation method, membrane filtration has also been used for the extraction of uranium ions. Wang et al.^[^ ^78^ ^]^ fabricated amidoximated cellulose fibers through grafting polyacrylonitrile and further amidoxime modification. Then the amidoximated cellulose fibers were combined with cellulose nanofibrils to fabricate a nonwoven membrane by vacuum filtration for dynamic uranium adsorption. The continuous dynamic extraction capacity of 1.22 mg g^−1^ at pH 8 was achieved by filtrating 10 L simulated seawater (U(VI): 9 mg L^−1^). We filtered amidoximated cellulose nanofibrils on a commercial nylon membrane to capture uranium ions through continuous flow (Figure 6E–G).^[^ ^16^ ^]^ After adsorption saturation, the membrane could be regenerated with a mixture solution of 1.0 M Na_2_CO_3_ and 0.1 M H_2_O_2_. More importantly, the membrane can remain an adsorption capacity of 437 mg g^−1^ in 20 ppm of uranium solution even after 5 adsorption‐desorption cycles. Mezzenga^[^ ^34f^ ^]^ combined amyloid nanofibrils and activated carbons to remove uranium efficiently (Figure 7H–J). After filtration, the concentration of U(VI) dropped from 1980 to 12.9 mg L^−1^, with a capturing efficiency of ∼99.4% (Figure 7J).

### 3.3 Chemical reduction of uranium ions

**FIGURE 8:**
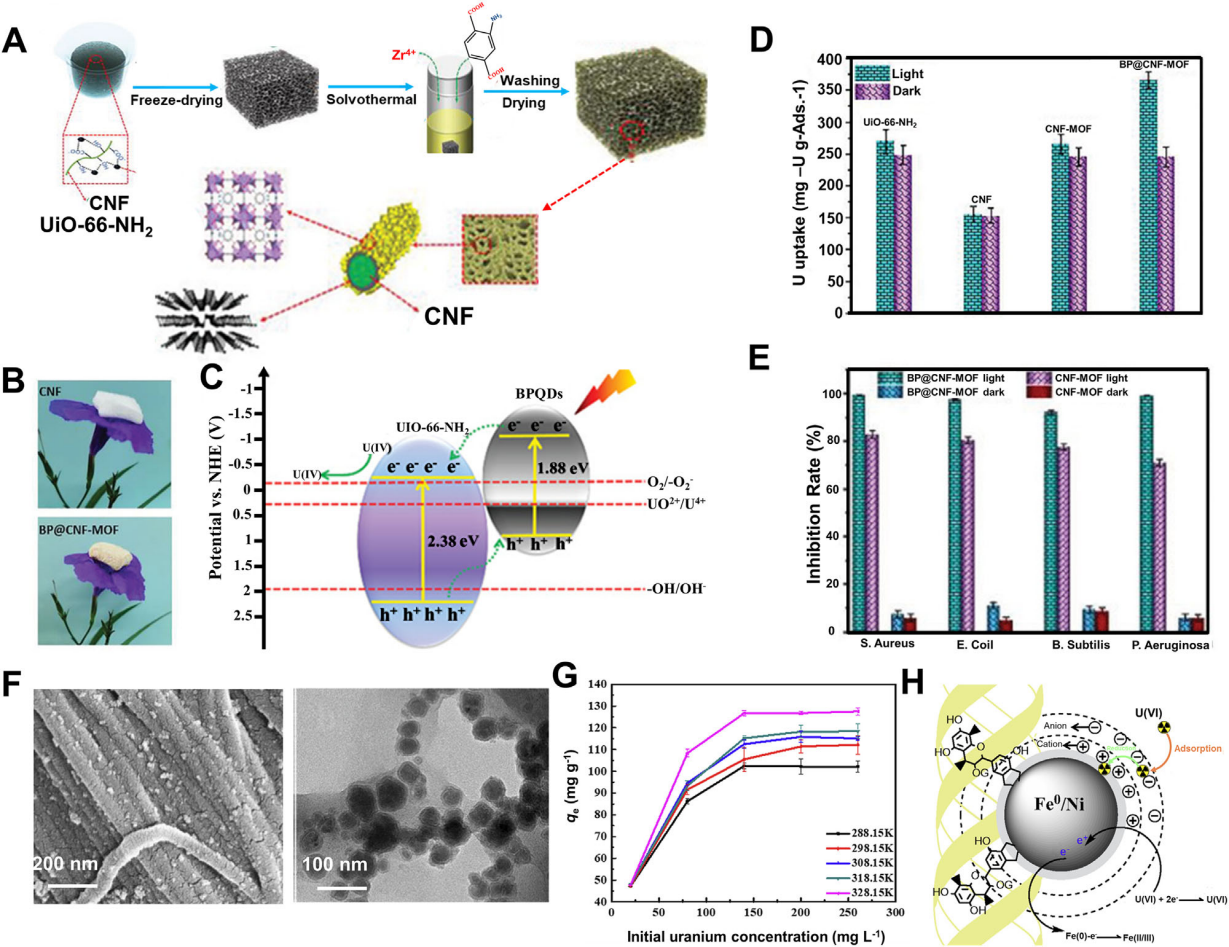
(A) Illustration of the hybrid aerogel (BP@CNF‐MOF) fabrication of black phosphorus quantum dots (BPQDs), cellulose nanofibril (CNF), and MOF (UiO‐66‐NH_2_). (B) Visual observation of pure CNF and BP@CNF‐MOF aerogels. (C) The proposed mechanism of the photocatalytic reduction of U(VI) under simulated sunlight irradiation. (D) Uranium uptake of UiO‐66‐NH_2_ powders, CNF aerogel, CNF‐MOF aerogel, and BP@CNF‐MOF aerogel under simulated sunlight irradiation and dark conditions. (E) Antibacterial activity of CNF‐MOF and BP@CNF‐MOF aerogel adsorbents under simulated sunlight irradiation and dark conditions. Reproduced with permission.^[^ ^34a^ ^]^ Copyright 2021, John Wiley and Sons. (F) SEM and TEM images of nano‐zero‐valent iron (NZFNP). (G) Effect of initial concentrations on U(VI) of adsorption performance of NZFNP. (H) The mechanism of adsorption and reduction for U(VI) elimination. Reproduced with permission.^[^ ^81^ ^]^ Copyright 2021, John Wiley and Sons

Chemical reduction of uranium ions into insoluble U(IV) like UO_2_ also provides a new way for extracting uranium from water solutions. Chen^[^ ^79^ ^]^ integrated MoS_2_ nanosheets into aerogels of bacterial cellulose. This Schottky heterostructure with a sulfur vacancy (S‐vacancy) could promote uranium extraction by photocatalytic U(VI) reduction. A high extracting efficiency up to 91% was achieved in a wide range of U(VI) concentrations from 10 to 60 mg L^−1^ under simulated sunlight irradiation. Chen et al.^[^ ^34a^ ^]^ decorated quantum dots of black phosphorus into aerogels of cellulose fibrils and further served as the template for in situ growing UiO‐66‐NH_2_ nanocrystals (Figure 8A,B). The composite aerogel exhibited excellent mechanical flexibility and high porosity (>98%) due to the strong binding interactions (e.g., coordination and H‐bonding) between cellulose nanofibrils and UiO‐66‐NH_2_ crystals. Black phosphorus quantum dots endowed the composites with superior photocatalytic activity to produce reactive oxygen species (ROS) (Figure 8C). ROS could photo‐catalytically reduce U(VI) into insoluble U(IV) of UO_2_. Under light irritation, their extraction capacity was ∼6.7 mg g^−1^ in real seawater with an efficiency increased by ∼55% compared to the case without light (Figure 8D). On the other hand, ROS could also kill marine bacteria (Figure 8E). This unique combination with anti‐bacterial property may prolong the service life of adsorbents in real seawater.^[^ ^65^, ^80^ ^]^

Liao^[^ ^81^ ^]^ introduced nanoparticles of zero‐valent Fe/Ni bimetals onto collagen nanofibers mediated by bayberry tannin (Figure 8F). As an effective reductant, Fe/Ni particles could reduce the adsorbed U(VI) into U(IV) which was confirmed by X‐ray photoelectron spectrum. Using these reductive nanofibers as extracting agent, the adsorption capacity can reach ∼130 mg g^−1^ (U(VI) concentration at 20 mg L^−1^) with an adsorption efficiency of 99.2% (Figure 8G,H).

### 3.4 Extraction of other nuclear metal ions

**FIGURE 9:**
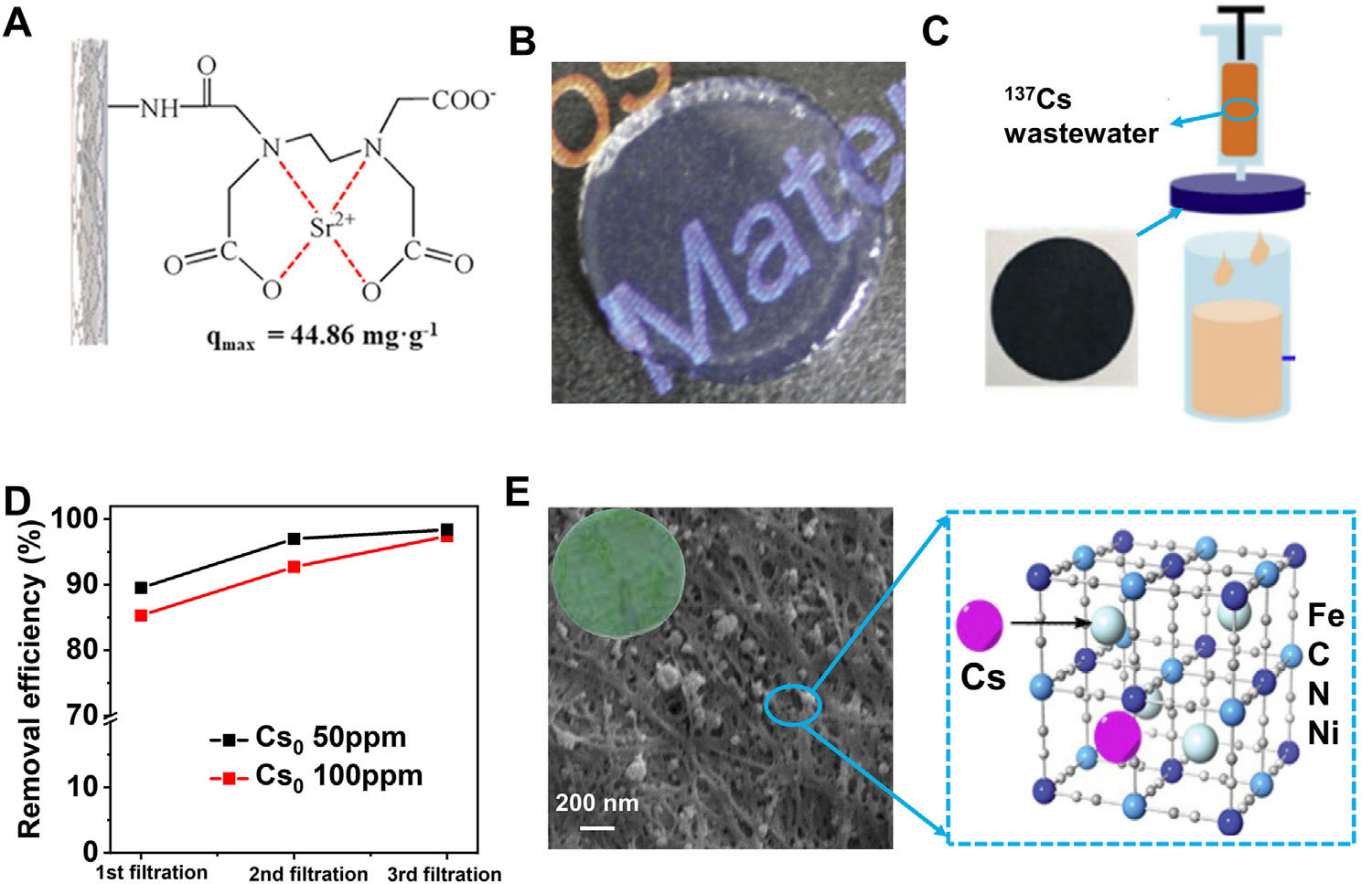
(A) The proposed mechanism of bacterial cellulose membrane modified with EDTA for Sr^2+^ adsorption. Reproduced with permission.^[^ ^83^ ^]^ Copyright 2018, Elsevier Ltd. (B) Optical image of hydrogel of TEMPO‐oxidized CNF. Reproduced with permission.^[^ ^84^ ^]^ Copyright 2013, Elsevier Ltd. (C) Illustration of the semicontinuous Cs^+^ adsorption apparatus and (D) Cs^+^ removal efficiency. Reproduced with permission.^[^ ^85e^ ^]^ Copyright 2020, Elsevier Ltd. (E) SEM image and photograph of bacterial cellulose membrane loaded with sodium nickel hexacyanoferrate nanoparticles. Reproduced with permission.^[^ ^85d^ ^]^ Copyright 2019, Elsevier Ltd

Thorium (Th), cesium (Cs), and strontium (Sr) are not only used in the nuclear industry, but also play an important role in the field of agricultural breeding, medical treatment, national defense, and laboratory proceedings.^[^ ^82^ ^]^ Cheng^[^ ^83^ ^]^ modified bacterial cellulose membranes with ethylenediaminetetraacetic acid, and possessed a maximum Sr^2+^ adsorption capacity up to 44.86 mg g^−1^ (Figure 9A). Isobe^[^ ^84^ ^]^ utilized TEMPO‐mediated oxidized cellulose nanofibrils to capture Cs^+^ with an uptake capacity of 133.8 mg g^−1^ (Figure 9B).

Many inorganic nanomaterials (e.g., nanobentonite, graphene oxide, Fe_3_O_4_, Prussian blue, and sodium nickel hexacyanoferrates) were introduced into cellulose nanofibrils as the low‐cost and efficient composite adsorbents.^[^ ^82^, ^85^ ^]^ For instance, Eun's group^[^ ^85e^ ^]^ embedded Prussian blue nanoparticles (size 5–20 nm) into a macroporous membrane of carboxymethyl cellulose nanofibrils for facile Cs^+^ capture via filtration (Figure 9C,D). The composite membrane presented an extraction capacity of 130 mg g^−1^, about 2.5‐fold greater than commercial Prussian blue nanoparticles, even with a low Prussian blue loading of 10 wt%.

Bacterial cellulose‐based Cs^+^ adsorbent was also fabricated via combining with sodium nickel hexacyanoferrates by Zhuang's group^[^ ^85d^ ^]^ (Figure 9E). The as‐prepared bio‐sorbent presented an excellent adsorption capacity of 175.4 mg g^−1^. Core–shell superparamagnetic magnetite nanoparticles adsorbent was also developed in Elrhman's group^[^ ^85b^ ^]^ via coating Fe_3_O_4_. The magnetic composite presented a maximum Th(IV) adsorption capacity of 880 mg g^−1^. Magnetic adsorbents have a great advantage for collection from water by magnetic field. Mezzenga^[^ ^86^ ^]^ found the hybrid membranes of amyloid nanofibrils and activated carbon could also extract technetium (Tc‐99m) and gallium (Ga‐68) by filtration with efficiencies above 99.8% in one single step of filtration.

## 4 EXTRACTION OF BATTERY‐RELATED METALS WITH BIOFIBROUS NANOMATERIALS

Battery‐related metals (e.g., Li Co, Ni, and Mn) have seen a sharp increase in demand with the fast development of the energy storage industry. With both the economic and environmental advantages, their recovery from retired batteries has also aroused great interest, particularly in the hydrometallurgical procedure. Meanwhile, almost all these metal ions could be found in seawater. And the amount of overall lithium inventory is reported to be approximately 2.6 × 10^11^ t.^[^ ^6b^ ^]^

### 4.1 Static adsorption of battery‐related metals ions

Nanocellulose has frequently been chemically modified to increase its adsorption property for Li ions. For example, Wahib^[^ ^87^ ^]^ impregnated date pits with cellulose nanocrystals with the presence of ionic liquid of 3‐formyl‐1‐methyl pyridinium iodide. The adsorbent presented a Li^+^ uptake of 99 mg g^−1^ at a concentration of 100 mg L^−1^ due to the existence of electrostatic interactions and H‐bonding between Li^+^ and less protonated adsorbent surface. Inorganic Li^+^ adsorbents, such as hydrogen manganese oxide^[^ ^88^ ^]^ and Li_4_Mn_5_O_12_ ionic sieve,^[^ ^89^ ^]^ were also loaded on cellulose fibrils. The ion exchanging, ion sieving, and ionic imprinting effects offered a high adsorption capacity and good selectivity.

**FIGURE 10:**
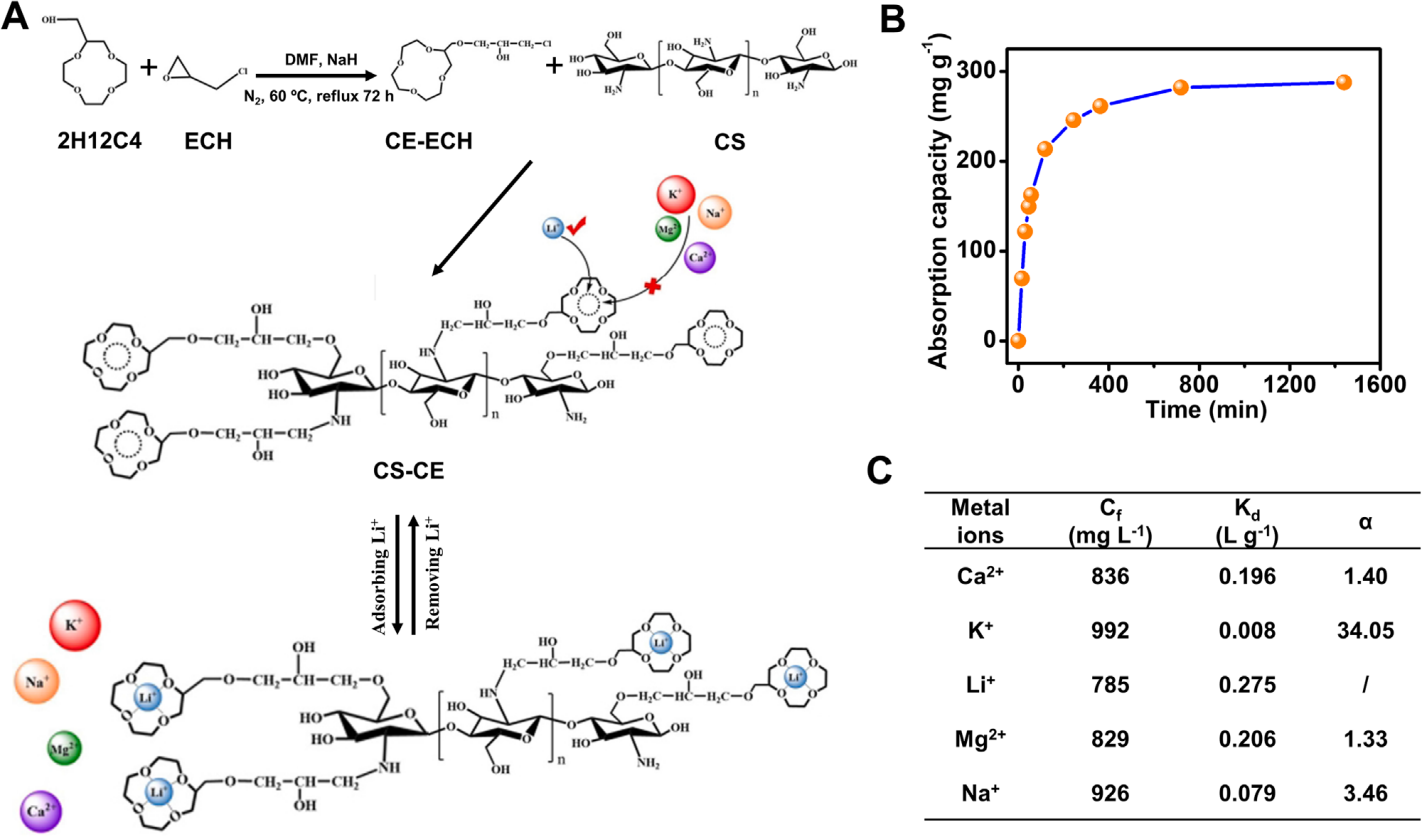
(A) Illustration of the reaction process of chitosan (CS) with 2‐(Hydroxymethyl)‐12‐crown 4‐Ether (2H12C4). ECH: epichlorohydrin. (B) Li^+^ adsorption kinetics with nanofibrous adsorbents of 2H12C4 modified chitosan (CS‐CE). (C) Selectivity of CS‐CE in Li^+^ adsorption. Reproduced with permission.^[^ ^90^ ^]^ Copyright 2021, Elsevier Ltd

Besides nanocellulose, Cheng et al.^[^ ^90^ ^]^ fabricated nanofibrous adsorbents of crown ether modified chitosan through evaporation‐induced phase separation (Figure 10A). With a specific surface area of about 111.6 m^2^ g^−1^, the aerogel‐like adsorbent acquired a maximum uptake of 297 mg g^−1^ within 3 h (Figure 10B). A considerable selectivity was achieved compared with other interfering metal ions (e.g., K^+^, Na^+^, Ca^2+^, and Mg^2+^) because the ionic radius of Li^+^ (0.68 Å) is approximate to the cavity diameter of 2‐(Hydroxymethyl)‐12‐crown 4‐Ether (2H12C4; 1.2–1.5 Å) (Figure 10C). When regenerating with 0.5 M HCl, the adsorbent remained the adsorption capacity after 5 adsorption–desorption cycles.

Through thermally induced phase separation, Zheng^[^ ^91^ ^]^ also prepared a nanofibrous membrane from crown ether functionalized graphene oxide, chitosan, and polyvinyl alcohol. The maximum Li^+^ adsorption capacity reached 168.5 mg g^−1^ at pH 7 due to its large specific surface area (∼101.5 m^2^ g^−1^). After 5 cycles, the material still retained 88.3% of the adsorption capacity.

Cobalt has received extensive attention as an important strategic metal, so the extraction of cobalt in water is also particularly important. Initially, Santoso et al.^[^ ^92^ ^]^ prepared cellulose nanocrystals with H_2_SO_4_‐hydrolysis. The adsorption isotherm was found to be well fitted with the Langmuir model, giving a maximum Co^2+^ extraction ability of 47.5 mg g^−1^. The adsorption proceeded exothermically, where the adsorption capacity declined with temperature. An optimal pH value of 6.6 was revealed since deprotonation of hydroxy groups enhanced their electrostatic interactions with Co^2+^. Narwade^[^ ^93^ ^]^ synthesized hydroxyapatite nanoparticles on cellulose nanofibrils, and obtained a maximum Co^2+^ uptake of 25 mg g^−1^. An extraction efficiency of 87% was also reached when the adsorbent dosage was 0.5 g L^−1^.

**FIGURE 11:**
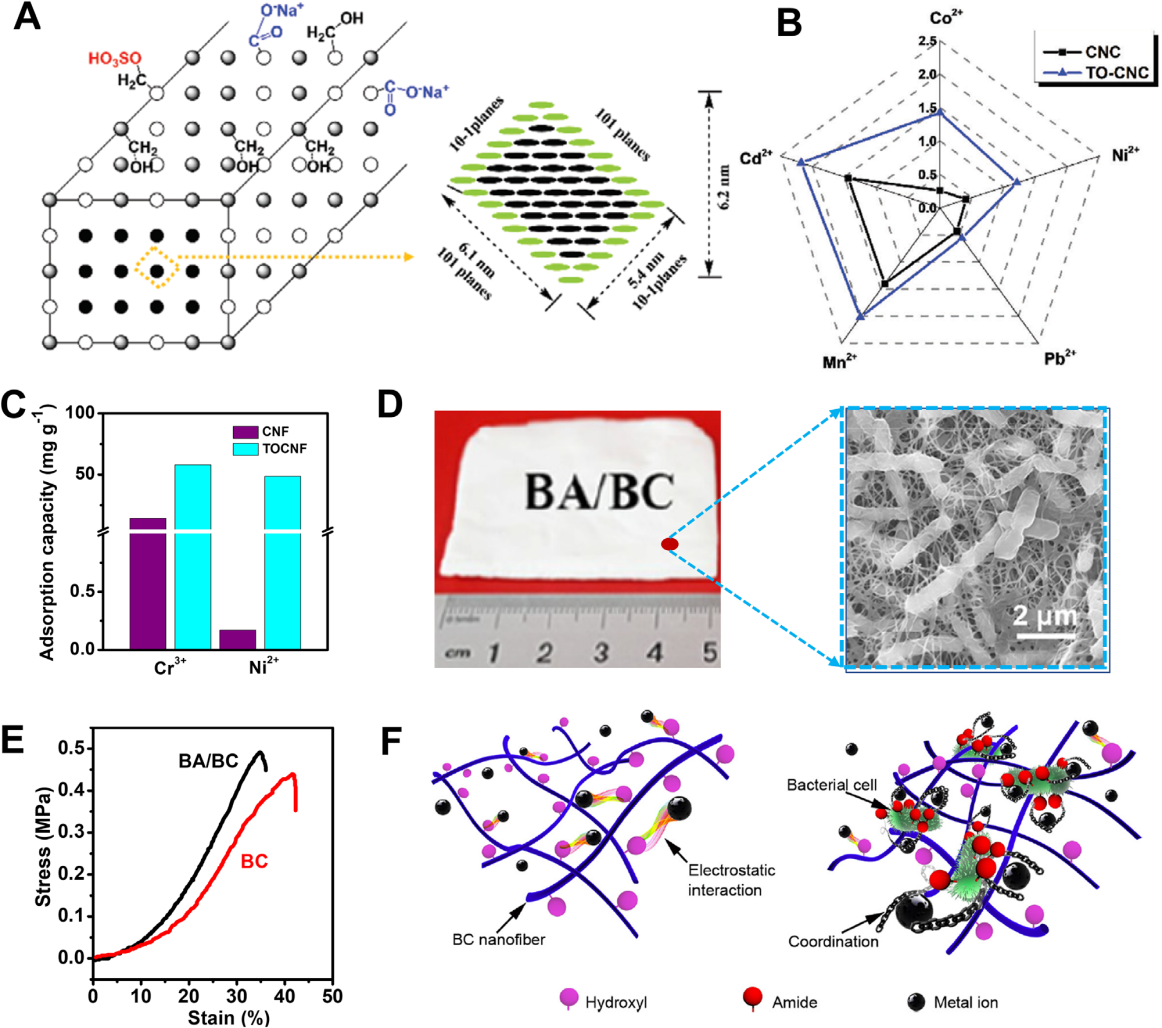
(A) The proposed crystalline model of pure cellulose nanocrystal (CNC) and TEMPO oxidation CNC (TO‐CNC). (B) Comparison of different metal ions adsorption on CNC and TO‐CNC. Reproduced with permission.^[^ ^94^ ^]^ Copyright 2021, John Wiley and Sons. (C) Adsorption of Cr^3+^ and Ni^2+^ onto mechanically disintegrated CNF and TOCNF. Reproduced with permission.^[^ ^96^ ^]^ Copyright 2014, Springer Science Business Media Dordrecht. (D) Optical and SEM images of bacteria/bacterial cellulose (BA/BC) hybrid aerogel. (E) Tensile–strain curves of pure (BC) and hybrid aerogels. (F) Adsorption mechanisms for the metal ions adsorption on the aerogels. Reproduced with permission.^[^ ^98^ ^]^ Copyright 2019, Elsevier Ltd

In comparison with H_2_SO_4_‐hydrolyzed cellulose nanocrystals, TEMPO oxidization could increase the adsorption capacity of Mn^2+^ 1.5–3.0 times through electrostatic adsorption,^[^ ^94^ ^]^ e.g., 111.1 mg g^−1^ (Figure 11A,B). Zhu^[^ ^95^ ^]^ endeavored to introduce Fe_3_O_4_ nanoparticles (∼15 nm in diameter) into bacterial cellulose matrix, through in situ synthesis and fermentation. With 33 wt% Fe_3_O_4_ loading, the nanocomposite showed a saturated magnetization of 41 emu g^−1^ and a related coercivity of 27 Oe, being potential for magnetic recovery. At the 200 mg L^−1^ ion concentrations, their adsorption capacities ranked as Pb^2+^ (50 mg g^−1^) > Mn^2+^ (25 mg g^−1^) > Cr^3+^ (20 mg g^−1^). When using sodium citrate as the regeneration eluent, the sequence of regeneration capacity was Mn^2+^ > Pb^2+^ > Cr^3+^.

Sehaqui et al.^[^ ^96^ ^]^ found the TEMPO oxidation could deliver cellulose nanofibrils with a Ni^2+^ extraction ability of 49 mg g^−1^ (Figure 11C), in which carboxyl groups offered negative charges and then attracted positively charged metal ions. Hokkanen^[^ ^97^ ^]^ introduced aminopropyltriethoxysilane groups into cellulose fibrils. With the presence of amino and/or hydroxyl groups, the maximum absorption capacity of Ni^2+^ was increased to 160.4 mg g^−1^. Without removing bacteria, Wan^[^ ^98^ ^]^ showed that bacterial cellulose had higher adsorption capacities of metals ions. The adsorption capacity of Ni^2+^ reached 4.5 mg g^−1^, being three times higher than that of pure bacterial cellulose (Figure 11D–F).

When loading carbonated hydroxyapatite on microfibrillated cellulose, Hokkanen^[^ ^99^ ^]^ reported that the maximum absorption capacity of Ni^2+^ was 118.6 mg g^−1^. 50% adsorption capacity was completed within 8 min and adsorption equilibrium was attained within 35 min. The fast rate could be attributed to the high density of functional groups of inorganic materials. In analog, Krivoshapkin^[^ ^100^ ^]^ loaded MnO_2_ nanoparticles on chitin nanocrystals, and presented a Ni^2+^ uptake of ∼114 mg g^−1^. The sorption mechanism should lie in ion‐exchange with porous MnO_2_ nanoparticles, and specific interaction and chelating with functional groups on chitin nanocrystals as well.

### 4.2 Dynamic filtration of battery‐related metals ions

Membrane filtration should an efficient separation method for strategic metal extraction. Guo^[^ ^101^ ^]^ further deposited carboxylated cellulose nanocrystals on a polyamide layer. With a water flux of 3.4 L m^−2^·h^−1^·bar^−1^, the resultant filtration membrane could separate divalent and multivalent ions from monovalent ions, and showed a rejection difference of ∼84% with the molar Mg^2+^/Li^+^ ratio at 60. To increase the adsorption capacity, Mathew^[^ ^45^ ^]^ added a zwitterionic polymer of poly(cysteine methacrylate) on a porous membrane produced from cellulose nanocrystals and TEMPO‐mediated oxidized cellulose nanofibrils. With the antifouling and antibacterial properties, poly(cysteine methacrylate) demonstrated the tendency to bind metal ions mainly through the thioether, amine, and carboxylate groups. The membrane exhibited a flux of 13.5 m^3^ h^−1^ m^−2^ bar^−1^ and a maximum Co^2+^ uptake of ∼42.6 mg g^−1^. Although filtration has high processing capability and considerable recyclability, this method has been rarely reported in the extraction of battery‐related metal ions. More research is highly required to improve their adsorption kinetics to match the high flux of nanofibrous membranes.

## 5 CONCLUSION AND PERSPECTIVE

The recent research progress of bio‐fibrous nanomaterials was reviewed as a novel class of nanostructured adsorbents for the extraction of strategic metal ions from seawater and industrial wastewater. Due to the unique combination of biological features (e.g., natural abundance, biodegradability, and biocompatibility) with large aspect‐ratio, high mechanical strength, and chemical diversity, bio‐fibrous nanomaterials have exhibited great promise in high adsorption capacity, fast dynamics, good processability, and sustainability.

A variety of “top‐down” and “bottom‐up” methods have been developed to exfoliate and regenerate biological nanofibrils from natural biomass such as wood, crustacea exoskeleton, silkworm cocoon silk, and animal bones. Chemical surface modifications have been frequently attempted with certain functional groups for binding affinity toward specific metal ions, for example, amidoxime toward U(VI), sulfhydryl toward Au(III), and crown ether toward Li^+^. Besides being used as adsorbents in dispersions, biological nanofibrils have also been assembled into bulk materials as diverse as fibers, aerogels, and membranes with adaptability to different adsorption forms of mooring in water, stationary bed, and filtration, respectively. These fibrous materials were generally constructed via physical interactions (e.g., H‐bonding and nanofiber entanglement) and chemical bonds (e.g., chemical cross‐linking) aiming to achieve high porosity, better hydrophilicity, high mechanical stability, and antifouling properties which are the successful features for real applications. In addition, composition with other functional materials like MOFs, MoS_2_, quantum dots, graphene, and oxides has also endeavored to acquire enhanced adsorption capacity, high selectivity, and photocatalytic activity.

Strategic metal ions were mainly targeted to noble metal ions (e.g., Au(III), Ag^+^ and Pt^2+^), nuclear metal ions (e.g., U(IV), Th(IV), and Cs^+^), and battery‐related metal ions (e.g., Li^+^, Ni^2+^, and Co^2+^), due to their indispensable functions in some important industries. Electrostatic attraction, chelation, chemical reduction, and nano‐sieving were addressed to enhance their adsorption capacities. Advances in eluant investigation offered a promising recovery avenue for both the adsorbates and fibrous adsorbents. Notably, biological nanofibrils with mild reductive activity and preferent capping capability (e.g., Au (111) crystal plane) were found to be able to reduce noble metal ions into 2D single crystals with large lateral size which showed huge potential in the application of microelectronics. The detailed study focused on adsorption isotherm, thermodynamics, dynamics, and pH effect could lay substantial foundations for practical applications of bio‐fibrous nanomaterials in extracting strategic metal ions. With these progresses, bio‐fibrous nanomaterials in the extraction of strategic metals should be a promising and exciting area.

However, there are still several hurdles that need to be overcome in the practical and large‐scale application of biofibrous nanomaterials for strategic metal ions extraction. First is the complex production and fabrication process which usually contains several steps of energy‐ and time‐consuming treatments like chemical modification and mechanical exfoliation. More research is needed to improve their preparation and chemical functionalization considering the aspects of economics, energy, and environmental costs. In addition, chemical functionalization would improve their adsorption capacity and selectivity, yet may influence their structural stability at the same time. Therefore, a balance should be taken into consideration during practical application in harsh chemical environment and violent hydrological conditions. Among these bio‐nanofibrils, cellulose nanofibrils exhibited high superiority for their low cost, abundance, and relative convenience in production and modification. Considering the economic efficiency, energy consumption, and environmental concern, cellulose nanofibrils have been considered to be closer to practical use. As for extraction methods, flow filtration could be more promising for metal ions capture compared with static adsorption, especially under high ion concentrations. High flux of nanofibrous membranes enables a higher processing capability with considerable recyclability. For low‐ion‐concentration environments like the natural sea, static adsorption may be more suitable despite the longer incubation periods that are usually required. Chemical reduction is mainly applicable to high valent metal ions with reductive biological nanofibrils, especially combined with additional reductants.

Furthermore, biodegradation is one of the important advantages of bio‐based adsorbents, in sharp contrast to synthetic adsorbents. To improve the serve life, antibacterial properties (e.g., quaternary ammonium group and guanidine group) are highly desired for these absorbents to extract strategic metal ions in the aquatic environment. Besides chemical modification, genetic engineering should be an effective alternative to produce biological nanofibrils (e.g., protein nanofibrils and bacterial cellulose nanofibrils) with these specific ligands.^[^ ^102^ ^]^ Electrochemical methods (e.g., electrochemical intercalation, electrochemical adsorption, and electrochemical reduction)^[^ ^14^ ^]^ should be a promising approach to offer biological nanofibrils with high extraction capability of metal ions, high selectivity, and high efficiency.

## CONFLICT OF INTEREST

The authors declare no conflict of interest.
